# Factors associated with low adherence to inhaled therapy in patients with chronic respiratory diseases: a cross-sectional study

**DOI:** 10.1186/s12890-025-03563-7

**Published:** 2025-02-27

**Authors:** Esther Ribes Murillo, Josep Ramon Marsal Mora, Marta Micol Bachiller, Leonardo Galván Santiago, Núria Nadal Braqué, Marta Ortega Bravo

**Affiliations:** 1 Unitat de Farmàcia, Gerència d’Atenció Primària i a la Comunitat de Lleida, Institut Català de la Salut, Rambla Ferran, 44, Lleida, 25007 Spain; 2 Fundació Institut Universitari per a la recerca a l’Atenció Primària de Salut Jordi Gol i Gurina (IDIAPJGol), Gran Via de les Corts Catalanes, 587, Barcelona, 08007 Spain; 3 RTI Health Solutions (RTI-HS), Av. Diagonal, 605, 9-1, Barcelona, 08028 Spain; 4 Unitat Docent, Gerència d’Atenció Primària i a la Comunitat de Lleida, Institut Català de la Salut, Rambla Ferran, 44, Lleida, 25007 Spain; 5 Centro de Atención Primaria C, Jose María Segura, Calasparra, Murcia, 30420 Spain; 6 Unitat de Farmàcia, Servei Català de La Salut, Av. Rovira Roure, 2, Lleida, 25006 Spain; 7 Direcció Assistencial d’Atenció Primària i a la Comunitat, Institut Català de La Salut, Gran Via de les Corts Catalanes, 587, Barcelona, 08007 Spain; 8 Centre d’Atenció Primària Almacelles. Carrer Melcior de Guàrdia, s/n, 25110 Almacelles. Gerència d’Atenció Primària i a la Comunitat de Lleida, Institut Català de la Salut, Lleida, Spain

**Keywords:** Asthma, Pulmonary Disease, Chronic Obstructive, Inhaler, Medication Adherence

## Abstract

**Background:**

Because of their high prevalence, chronic respiratory diseases, like asthma and chronic obstructive pulmonary disease, represent main public health problems. They are mainly treated through inhaled therapy. There is low adherence to such therapy, resulting in poor control of chronic respiratory diseases. However, more research is needed on the association of several factors with low adherence.

The purpose of this study was to estimate the association of age, sex, type of drug, and frequency of administration with low adherence to inhaled therapy. In order to do this, we performed a cross-sectional study.

**Methods:**

We selected all patients treated with long-acting anticholinergics (LAMA), long-acting β2-adrenergics (LABA), LAMA/LABA, or inhaled corticosteroid (ICS)/LABA in the Health Area of Lleida on 16 March 2017. For each treatment, we determined the percentage of patients showing low adherence to therapy (less than 50%), calculated as drug boxes collected from the pharmacy with respect to the prescribed ones. Then, we analysed the association of age, sex, type of drug, and frequency of administration, with low adherence to therapy through a multivariate linear model.

**Results:**

11,128 people had electronic prescriptions for one of the inhaled therapy; of them, 24.6% (2,741) showed low adherence. The highest percentage of people with low adherence was found among young patients and women. Women 25–34 years of age included the highest percentage of patients with low adherence. As for drugs, the highest percentage of patients with low adherence was found among the ones treated with LABA and ICS/LABA. Finally, a higher percentage of patients with an administration frequency of 12 h presented low adherence, in comparison with patients treated every 24 h, in general and in the LABA and ICS/LABA groups.

**Conclusions:**

The differences that we observed in adherence to inhaled therapy according to the different factors analysed should be considered when managing chronic respiratory diseases and their impact on patients’ clinical burden, quality of life, and costs for the health system.

## Background

Chronic respiratory diseases affect the airways and other structures of the lungs. The most common are asthma and chronic obstructive pulmonary disease (COPD). Approximately, half a billion people live with asthma and COPD. Together, these two conditions cause almost 4 million deaths every year. More than 1 million of these deaths occur in people under 70. Moreover, COPD is the third leading cause of death worldwide [1].

GINA [2] guidelines for asthma and GOLD [3] and GesEPOC [4] guidelines for COPD state that the primary objective of their treatment is to reduce the symptoms, and the frequency and severity of exacerbations. They establish that inhaled therapy (IT) is the main pharmacological treatment, and that, by following it correctly, not only the symptoms and the frequency and severity of exacerbations can be reduced, but also exercise tolerance and overall health can improve.

Adherence is defined as the extent to which a person’s behaviour (taking a medication in this case) corresponds with the agreed recommendations from a healthcare provider [5]; however, there is no clear consensus on the definition of poor adherence [6]. In the majority of studies, adherence is considered adequate when above 80% [7]. In chronic pathologies, the percentage of patients with adherence to therapy greater than 80% ranges from 75% for hypertension, hyperlipidaemia, osteoporosis, multiple sclerosis and cancer, to 33% for asthma and COPD. Moreover, the average adherence to IT for chronic respiratory diseases ranges from 25 to 68% [8]. Therefore, some studies have used the cut-off point of 50% for low adherence in patients with chronic respiratory diseases [9, 10]. Such low adherence among patients with asthma and COPD represents a major public health problem, affecting how the diseases are controlled, and resulting in the prescription of unnecessary treatments and an increase in hospital visits and admissions [11]. In turn, this implies high costs for healthcare in many countries. In the USA, it is estimated that the annual cost of low adherence in patients with chronic diseases amounts to 300 billion dollars, with COPD being among the ones with the lowest adherence rates [11].

Several patient-related factors influence adherence to inhaled therapy in obstructive lung diseases. One notable factor is smoking, which has been consistently associated with poor adherence to prescribed inhalation therapies. Smokers may have a higher likelihood of non-adherence, possibly due to a combination of factors such as disease progression, reduced perceived treatment effectiveness, and lifestyle behaviours. Smoking could exacerbate the symptoms of diseases like asthma and COPD, leading to decreased motivation for treatment adherence [12]. In addition to smoking, other factors such as age, comorbidities, regimen complexity, adverse effects, level of knowledge of the disease, poor doctor-patient communication, and cost of medications also play significant roles in determining adherence to therapy [13]. Research has shown that these factors can interact in complex ways, highlighting the need for further research to understand their relative impact on adherence and treatment outcomes.

## Methods

The objective of this study was to evaluate the association of low adherence to inhaled therapy with age, sex, type of drug, and frequency of administration. To this aim, we performed a cross-sectional study.

### Study design

We performed an observational cross-sectional study in the Health Area of Lleida based on anonymous data from electronic prescriptions provided by the Catalan Health Service on 16 March 2017. This data included drug, brand, dose, frequency, and duration of therapy, as well as the number of available boxes for the patient to collect from the pharmacy and the number of boxes collected at the moment of the study.

### Sample, setting

The study involved patients in the Lleida Health Area. It focused on individuals who had prescriptions for inhaled therapies (IT) that included one of the following drugs or combinations: a long-acting anticholinergic (LAMA), a long-acting beta-2 agonist (LABA), a LAMA plus LABA combination, and an inhaled corticosteroid (ICS) plus LABA, as commercialized in Spain at the time of the study (Table 1).

**Table 1:** Drugs included in the study and their frequency of administration

Pharmacological family	Drug	Frequency of administration (hours)
LAMA	Aclidinium	12
	Tiotropium	
	Glycopyrronium	24
	Umeclidinium	
LABA	Salmeterol	12
	Formoterol	24
	Indacaterol	
	Olodaterol	
LAMA/LABA	Aclidinium/formoterol	12
	Glycopyrronium /indacaterol	24
	Tiotropium/olodaterol	
	Umeclidinium/vilanterol	
ICS/LABA	Fluticasone/salmeterol	12
	Beclometasone/formoterol	
	Budesonide/formoterol	
	Fluticasone/formoterol	
	Fluticasone/vilanterol	24

*LABA* long-acting beta-2 agonist, *LAMA* long-acting anticholinergic, *ICS* inhaled corticosteroid

### Inclusion–exclusion criteria

Patients with an electronic prescription for at least one of the IT with the drugs or combination of them included in the study. Patients with prescriptions for ICS alone were excluded from the study, as these patients may have seasonal allergic asthma, which could confound the adherence data.

### Statistical analysis

Adherence to IT was calculated by dividing the number of boxes collected from the pharmacy by the number of boxes prescribed by the physicians during the validity of the electronic prescription at the time of the study. Numerical variables were expressed as mean and standard deviation, and categorical variables were expressed as absolute and relative frequencies. Differences in the proportion of patients with low adherence were calculated by age, sex, type of drug, and frequency of administration, using the Z-test with a 95% confidence interval (95% CI). The association of different variables with adherence was evaluated through a multivariate linear model, using the percentage of adherence as the response variable and the rest of the variables as predictors. Regression coefficients, Odds Ratios, and 95% CI were calculated.

### Ethical considerations

This study was approved by the ethics and clinical research committee at the Institut d’Investigació IDIAP Jordi Gol under the code P18/012. The study was conducted in accordance with the principles of the Declaration of Helsinki. The variables in the electronic prescription database provided by the Catalan Health Service were processed anonymously and we fully guaranteed confidentiality, as established by the national law and the Regulation 2016/679 of the European Parliament and of the Council on the protection of natural persons with regard to the processing of personal data, and to the free movement of such data. Accordingly, it was not necessary to ask participants for their informed consent.

## Results

We identified 11,128 patients, the average age was 66.0 ± 19.1 years and 44.7% (4,970) were female. The detailed distribution of sex, age, type of drug, and frequency of administration for patients with at least one of the IT included in the study is shown in Table 2.

**Table 2:** Sex, age, type of drug and frequency of administration of patients with at least one of the IT included in the study

		N	%
**Sex**	Men	6,158	55.3%
	Women	4,970	44.7%
**Age group (years old)**	0–14	248	2.2%
	15–24	253	2.3%
	25–34	328	2.9%
	35–44	683	6.1%
	45–54	1,114	10.0%
	55–64	1,709	15.4%
	65–74	2,487	22.3%
	75–84	2,660	23.9%
	85–94	1,535	13.8%
	> 94	111	1.0%
**Type of drug**	LAMA	3,569	32.1%
	LABA	1,809	16.3%
	LAMA/LABA	833	7.5%
	ICS/LABA	7,127	64.0%
**Frequency of administration**	Every 12 h	8,719	78.4%
	Every 24 h	4,175	37.5%

**Fig. 1:**
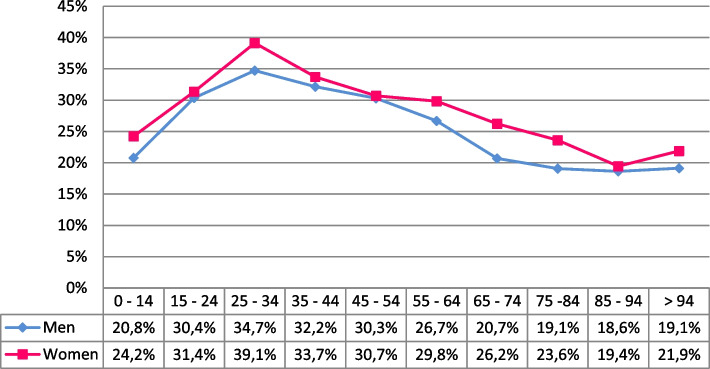
Percentage of patients with low adherence according to sex and age group

A percentage of 24.6% of the patients (2,741/11,128) showed low adherence. When stratifying by age, we found the highest percentage of patients with low adherence among 25- to 34-year-olds (36.9%). As for sex, 23.0% of men (1,417/6,158) and 26.6% of women (1,324/4,970) presented low adherence (Odds Ratio = 1.21 (1.11–1.32), *p* < 0.001). Considering both age and sex, the highest percentage of patients with low adherence was among 25- to 34- year-old women (39.1%; 95% CI: 31.6%—46.7 Finally, a statistically significant higher percentage of women showed low adherence, in comparison to men, in age groups 65 to 74 years (Odds Ratio = 1.36 (1.13–1.65), *p* < 0.001) and 75 to 84 years (Odds Ratio = 1.31 (1.09–1.58), *p* = 0.005). This gender difference in adherence may be related to the distinct clinical course and characteristics of airway diseases in men and women. For example, research has shown that women have a higher prevalence of asthma, particularly in older age groups, which is associated with poorer asthma control. Female patients with asthma tend to experience more comorbidities, such as obesity, hypertension, and thyroid diseases, which can exacerbate the condition and potentially influence treatment adherence. Studies have highlighted that being female, along with the presence of conditions like gastroesophageal reflux disease, is linked to poorly controlled asthma, thus leading to worse adherence rates in this population (Fig. 1).

As for the type of drug, the LABA group had the highest percentage of patients with low adherence (26.6%), and the LAMA/LABA group had the lowest percentage of patients with low adherence (8.8%) (Table 2). The LAMA group had fewer patients with low adherence than the LABA and ICS/LABA groups (*p* < 0.001), and more patients with low adherence than the LAMA/LABA group (*p* < 0.001). Also, the LABA group had more patients with low adherence than the LAMA/LABA and ICS/LABA groups (*p* < 0.001). Finally, the LAMA/LABA group had fewer patients with low adherence than the ICS/LABA group (*p* < 0.001) (Table 3).

**Table 3:** Differences in the proportion of patients with low adherence according to type of drug

Treatment 1	n/N	%	Treatment 2	n/N	%	Difference (95% CI)	Odds Ratio (95% CI)
LAMA	669/3568	18.8%	LABA	482/1809	26.6%	−7.8%^*^ (−10.3%; −5.5%)	0.64 (0.56–0.73)
			LAMA/LABA	73/832	8.8%	10.0%^*^ (7.7%; 12.3%)	2.39 (1.86–3.09)
			ICS/LABA	1710/7123	24.0%	−5.2%^*^ (−6.9%; −3.6%)	0.73 (0.66–0.81)
LABA	482/1809	26.6%	LAMA/LABA	73/832	8.8%	17.8%^*^ (15.1%; 20.7%)	3.78 (2.91–4.91)
			ICS/LABA	1710/7123	24.0%	2.6%^**^ (0.4%; 4.9%)	1.15 (1.02–1.29)
LAMA/LABA	73/832	8.8%	ICS/LABA	1710/7123	24.0%	−15.2%^*^ (−17.4%; −13.1%)	0.30 ( 0.24–0.39)

*LABA* long-acting beta-2 agonist, *LAMA* long-acting anticholinergic, *ICS* inhaled corticosteroid

^*^p < 0.001

^**^p = 0.023

According to the frequency of administration of the drugs, a higher percentage of patients treated every 12 h had low adherence, in comparison to those treated every 24 h. Specifically, the proportion of patients with low adherence was 25.1% in the 12 h group, and 17.1% in the 24 h group, with a difference of 8.0% (95% CI: 6.6%−9.5%, *p* < 0.001). Finally, considering the type of drug and frequency of administration together, in the LABA and ICS/LABA groups, we observed a significant higher percentage of patients with low adherence among the ones receiving the treatment every 12 h than among the ones receiving it every 24 h (*p* < 0.001) (Table 4).

**Table 4:** Patients with low adherence according to the frequency of administration and the type of drug

	**n/N**	**%**	**Difference (95% CI)**	**Odds Ratio (95% CI)**	***p***
**Patients with at least one IT**	2,741/11,128	24.6%			
**Every 12 h**	2,186/8,719	25.1%	8.0% (6.6%; 9.5%)	1.63 (1.48–1.79)	< 0.001
**Every 24 h**	712/4,175	17.1%			
**LAMA 12 h**	78/372	21.0%	2.5% (−1.9%; 6.8%)	1.17 (0.90–1.53)	0.264
**LAMA 24 h**	591/3,197	18.5%			
**LABA 12 h**	446/1,584	28.2%	12.2% (6.9%; 17.4%)	2.06 (1.42–2.99)	< 0.001
**LABA 24 h**	36/225	16.0%			
**LAMA/LABA 12 h**	10/138	7.2%	−1.9% (−6.6%; 3%)	0.78 (0.39–1.57)	0.460
**LAMA/LABA 24 h**	63/695	9.1%			
**ICS/LABA12h**	1,680/6,876	24.4%	12.4% (8.3%; 16.6%)	2.38 (1.62–3.50)	< 0.001
**ICS/LABA24h**	30/251	12.0%			

## Discussion

This cross-sectional study reveals that around 25% of patients with chronic respiratory diseases had an adherence to IT lower than 50% in the Health Area of Lleida on 16 March 2017. We found the highest percentage of patients with low adherence among younger people, women, patients treated with LABA and ICS/LABA, and those presenting higher administration frequency.

Using the same cut-off point of 50%, Broder et al. [10] found that, between 1 January 2003 and 30 June 2004 in United States, 60.5% of patients with asthma showed low adherence to IT with ICS/LABA. Moreover, Humenberger et al. [9] found that, in 2012 in Upper Austria, 42.6% of patients with COPD and treated with LAMA, LABA, LAMA/LABA, ICS/LABA, or ICS/LABA/LAMA had an adherence lower than 50% after hospital discharge and after 24-month follow-up. These percentages are higher than the one we found, and this may be due to differences in pathologies, types of drug analysed, characteristics of the patients, and types of analysis. In particular, in the study by Broder et al. [10], all the patients had asthma; in the study by Humenberger et al. [9], all had COPD; and in ours, no distinction was made according to the diagnosis. As far as we know, there is no previous study assessing adherence to inhaled therapy in general. Moreover, in the study by Broder et al. [10], the only treatment analysed was one ICS/LABA; in the study by Humenberger et al. [9], 77% of the patients were treated with ICS/LABA/LAMA and, in our study, we analysed LAMA, LABA, and the combinations LAMA/LABA and ICS/LABA. At the time of our study, no combination of ICS/LABA/LAMA in a single device had been marketed. Despite the differences between the three studies, the percentage of patients with low adherence is considerable. Furthermore, previous literature emphasizes that low adherence to IT can significantly impact disease outcomes, such as exacerbations and disease control status. For instance, Makelä et al. observed that poor adherence was associated with worse health outcomes and higher healthcare costs in both asthma and COPD populations [13]. Similarly, Humenberger et al. highlighted the clinical implications of low adherence, particularly regarding exacerbation risk, reinforcing the need for targeted strategies to improve adherence rates in these patient groups [9]. These points out the importance to establish measures to improve the adherence to IT to control the symptoms of chronic respiratory diseases and avoid their exacerbation. In contrast to all these studies, Izquierdo et al. [14] found that average adherence (measured as the ratio of the doses collected from the pharmacy and the number of days covered according to product labelling) to LAMA in patients with COPD was greater than 100%. Such discrepancy could be due to differences in electronic prescription systems. In particular, the system used in our Health Area only permits patients to collect the prescribed amount, never exceeding 100%.

We observed that patients older than 75 years had the best adherence. Similarly, in their study, Bender et al. observed that adherence, calculated as the total number of days of inhaled ICS/LABA supply in a year, was higher in the group of elderly patients (> 70 years) [15]. One reason could be that at these ages, patients are followed up by caregivers that prepare the medication and supervise them.

Considering sex, like in previous studies [6, 10], we show that a higher percentage of women had low adherence to IT in comparison to men. On the contrary, in a study by Izquierdo et al. [14], no differences in the percentage of women and men with low adherence were observed. In our study, we observed that a higher percentage of women had low adherence to inhaled therapy compared to men, with the highest percentage among women aged 25 to 34 years. This aligns with the observations by Karadoğan et al., suggesting that sex-specific factors may influence asthma control and medication adherence [16]. This result is surprising since a main factor associated with low adherence is polypharmacy, which is not expected young women. A study carried out by Haupt et al. [17] show similar results, whereas Broder et al. [10] found that only 15.1% of patients (both men and women) in the same range of age (25–34 years old) had an adherence to IT lower than 50%. The explanation for low adherence in young women, in whom polypharmacy is not expected, may lie in anthropological factors that complicate self-care. Dorothea Orem’s theories on self-care deficits and the importance of self-care for individuals affected by diseases have been highlighted by later authors [18, 19]. Additionally, women’s health is influenced by multiple dimensions of their lives, such as reproductive function, biological realities, gender inequality, and the social context in which they live and work. These factors can affect their habits and daily activities, which play a significant role in self-care [20, 21]. Moreover, beliefs about medication also impact adherence. In particular, pregnant women often express concerns about the safety of drugs, which can hinder adherence. While some women perceive the benefits of inhaled corticosteroids (ICS) to outweigh the risks, many overestimate the teratogenic risks associated with these medications, leading to reluctance to use them [22–24]. Such beliefs, alongside other sociocultural factors, can contribute to lower medication adherence in this demographic, emphasizing the need for targeted educational interventions and support strategies to address these barriers effectively.The observed discrepancies in adherence across demographic groups underscore the need for individualized approaches to improve adherence, as highlighted in studies such as those by Makelä et al. and Ismaila et al., where poor adherence was linked not only to clinical outcomes but also to increased healthcare costs and worse disease control in COPD patients [25].

Considering the type of drug used, we found the highest percentage of patients with low adherence among the ones taking LABA or ICS/LABA. A previous study by Foden et al. [26] found a highest percentage of patients with low adherence among the ones treated with ICS, in comparison to patients only treated with ICS/LABA. However, ICS alone may be used seasonally, which may falsify adherence. For this reason, we excluded patients treated with ICS from our study.

In terms of frequency of administration, our study show that a higher percentage of patients using IT every 12 h had low adherence, in comparison to those using IT every 24 h. A similar observation was made in previous studies in patients taking medications for other chronic diseases [27, 28]. Few other studies have explored the correlation between frequency of administration and adherence to IT. In agreement with ours, a study [29] in patients with asthma revealed worse adherence among patients who received doses every 6 h, in comparison to those who received them every 12 h. Another study found that adherence was worse in asthmatic patients treated every 12 h in comparison to those treated every 24 h with ICS/LABA. Our findings align with prior evidence suggesting that simplifying administration schedules can improve adherence. As noted by Ismaila et al., better adherence to combination therapies like tiotropium and ICS/LABA has a direct impact on reducing exacerbation rates and improving overall disease management in COPD [26].

Finally, we combined the analysis of adherence to IT by type of drug and the one by frequency of administration. As in a study by Izquierdo et al. [14], we found no statistically significant differences in the percentage of patients showing low adherence between the ones treated with LAMA every 12 h and the ones treated every 24 h.

One limitation of our study is not differentiating between diagnoses or type of device, since this information was not available in the database. But at the same time, it is a strength as it allows the assessment of adherence to IT globally in a population cohort with respiratory pathology, and regardless of the device used.

Therefore, more research is needed to assess the correlation of specific diseases or type of device with the adherence, and its possible interplay with the rest of variables here studied.

## Conclusions

One in four patients collected less than 50% of the IT prescribed and the percentage of patients with low adherence changed according to age, sex, treatment, and administration frequency. These differences should be considered when managing chronic respiratory diseases to avoid possible consequent exacerbation of the illness and costs for the health system.

## Data Availability

The datasets used and/or analysed during the current study are available from the corresponding author on reasonable request by the editors.

## Abbreviations

COPD: Chronic obstructive pulmonary disease
GesEPOC: Spanish COPD Guidelines
GINA: Global Initiative for Asthma
GOLD: Global Initiative for Chronic Obstructive Lung Disease
ICS: Inhaled corticosteroid
IT: Inhaled therapy
LABA: Long-acting β2-adrenergics
LAMA: Long-acting anticholinergics
